# Limited intervention improves technical skill in focus assessed transthoracic echocardiography among novice examiners

**DOI:** 10.1186/1472-6920-12-65

**Published:** 2012-08-03

**Authors:** Christian Alcaraz Frederiksen, Peter Juhl-Olsen, Dorte Guldbrand Nielsen, Berit Eika, Erik Sloth

**Affiliations:** 1 Department of Anesthesiology and Intensive care, Aarhus University Hospital, Brendstrupgaardsvej 100, 8200 Aarhus, Denmark; 2 Unit for Medical Education, Faculty of Health Sciences, Aarhus University, Aarhus, Denmark; 3 Institute of Clinical Medicine, Faculty of Health Sciences, Aarhus University, Aarhus, Denmark

**Keywords:** Point-of-care, Bedside, Ultrasound, Echocardiography, Learning

## Abstract

**Background:**

Previous studies addressing teaching and learning in point-of-care ultrasound have primarily focussed on image interpretation and not on the technical quality of the images. We hypothesized that a limited intervention of 10 supervised examinations would improve the technical skills in Focus Assessed Transthoracic Echocardiography (FATE) and that physicians with no experience in FATE would quickly adopt technical skills allowing for image quality suitable for interpretation.

**Methods:**

Twenty-one physicians with no previous training in FATE or echocardiography (Novices) participated in the study and a reference group of three examiners with more than 10 years of experience in echocardiography (Experts) was included. Novices received an initial theoretical and practical introduction (2 hours), after which baseline examinations were performed on two healthy volunteers. Subsequently all physicians were scheduled to a separate intervention day comprising ten supervised FATE examinations. For effect measurement a second examination (evaluation) of the same two healthy volunteers from the baseline examination was performed.

**Results:**

At baseline 86% of images obtained by novices were suitable for interpretation, on evaluation this was 93% (p = 0.005). 100% of images obtained by experts were suitable for interpretation. Mean global image rating on baseline examinations was 70.2 (CI 68.0-72.4) and mean global image rating after intervention was 75.0 (CI 72.9-77.0), p = 0.0002. In comparison, mean global image rating in the expert group was 89.8 (CI 88.8-90.9).

**Conclusions:**

Improvement of technical skills in FATE can be achieved with a limited intervention and upon completion of intervention 93% of images achieved are suitable for clinical interpretation.

## Background

Point-of-care (POC) ultrasonography [1-4] is rapidly expanding throughout acute medicine, intensive care medicine, and the pre-hospital setting.

In the cardiopulmonary area the Focus Assessed Transthoracic Echocardiography (FATE) [5] protocol combines POC echocardiography with imaging of the pleura. The FATE protocol consists of a set of predefined scanning positions in which the examiner is able to exclude obvious pathology, assess wall thickness and determine cardiac dimensions essential for the evaluation of preload / afterload and myocardial function. The existing experience with FATE is limited [5-7], and little is known about technical image quality and performance of POC in the hands of inexperienced examiners with no formal training. A number of studies have looked at inexperienced examiners within the domain of transthoracic echocardiography [8] and POC [9-11] echocardiography. However, these studies have primarily focussed on image interpretation and not on the technical image quality. Digital simulation has also received some attention, but primarily in the field of trans-oesophageal echocardiography and with focus on technical skills [12,13].

The effect of previous clinical experience and visual spatial ability could possibly affect the ability to learn POC ultrasound. However, we have not found studies focussing on the prerequisites of the examiner nor did we identify studies describing the nature and amount of training needed in order to perform a FATE examination with sufficient technical image quality.

With the aim of describing the required technical skills for adequate imaging of the heart according to the FATE protocol and the impact of visual spatial ability, we hypothesize that among novice examiners, a limited intervention of 10 supervised examinations could improve the technical skills in FATE measured as the percentage of images suitable for interpretation.

## Methods

### Study population

The study population comprised two groups of participants: a study group of twenty-one physicians (9 females) with no previous training in FATE or echocardiography (Novices) and as a reference group three examiners with more than 10 years of experience in echocardiography (Experts). The experts had done far beyond 1000 studies and perform focused studies as well as standard echocardiography on a daily basis. Mean age of novices was 32.3 years (30.6-34.0) and the mean clinical experience was 4.0 years (2.6-5.3). Approximately half of the participants (11 (52%)) had previous experience with ultrasonography (nerveblocks, vascular access etc.). All subjects were recruited by written invitation and participation was voluntary and without compensation.

The study was exempt from formal ethical approval according to the institutional review board. Regardless, we made considerable efforts to protect the interests of the participants: participation was voluntary, we analysed the data anonymously, informed the participants about the study in writing, and notifying them of their right to withdraw consent at any time. None of the interviewees withdrew their consent. We also encouraged the participants to contact the researchers if they had any concerns or questions.

### Echocardiography equipment

A Vivid E9 (GE Healthcare, Horten, Norway) ultrasound system equipped with a M5S transducer (1.5 – 4.5 MHz) was used for all baseline and evaluation examinations by novices as well as experts. A Vivid Q (GE Healthcare, Horten, Norway) system equipped with a M4S transducer (1.5 – 4.0 MHz) was used for the 10 supervised examinations. Both systems can be considered as high-end equipment.

### Subjects

Two healthy adult volunteers served as subjects for examination before and after intervention. For each participant, ten patients with or without cardiopulmonary pathology from the medical department of the institution served as cases for the intervention.

### Data acquisition

Novices received an initial introduction (2 hours) including a theoretical review of basic ultrasound theory, review of the examination protocol, introduction to the ultrasound equipment and general information about the study. Following the introduction, baseline examinations were performed on the two healthy volunteers. The novices were asked to perform the examinations as described in the FATE protocol and were allowed 90 seconds to obtain each view. For each volunteer, the participants were asked to record a total of six standard views consisting of the following: 1) Subcostal 4-chamber view, 2) Apical 4-chamber view, 3) Parasternal long axis view, 4) Parasternal short axis view, 5) Pleura right side, 6) Pleura left side. A supervisor was present at all times with the purpose of assisting novices in adjustment of gain, depth, and angle, but only if requested. The supervisor recorded whether these adjustments were asked for and whether the probe orientation was correct, by checking the position of the orientation marker. All images were stored digitally for subsequent offline analysis.

Following the FATE examinations all novices were subjected to a mental rotation test in order to assess visual spatial ability as described by Vandenberg and Kuse [14,15].

**Figure 1:**
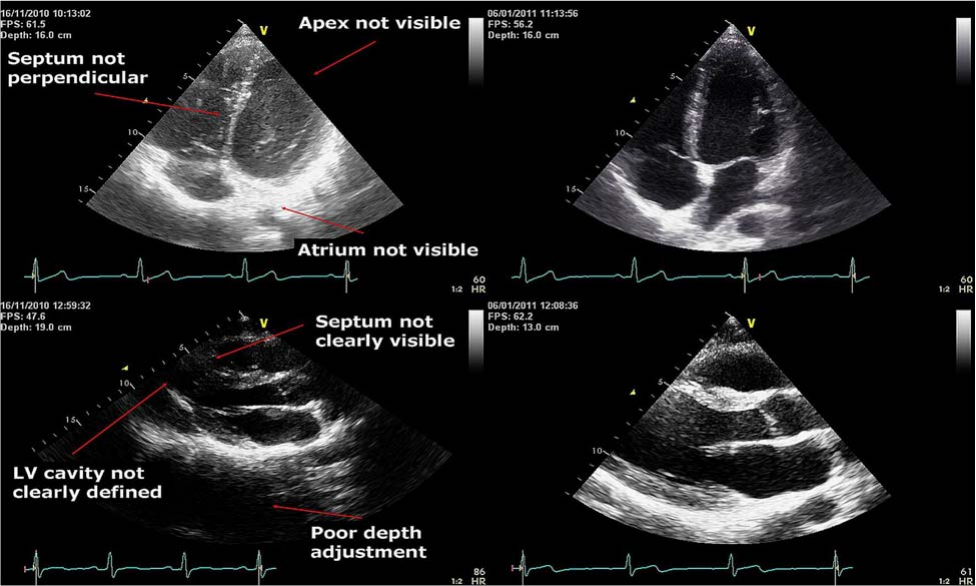
**Actual examples of image quality before and after intervention.*** Upper left panel:* Apical 4-chamber view at baseline. *Upper right panel:* Apical 4-chamber view at evaluation. *Lower left panel:* Parasternal long axis view at baseline. *Lower right panel:* Parasternal long axis view at evaluation.

After baseline examinations all physicians were scheduled to a separate intervention day comprising ten supervised FATE examinations. Information and supervision were done one-on-one in a uniform manner by only one supervisor with a focus exclusively upon transducer movement and technical image quality. For effect measurement a second examination (evaluation) of the same two healthy volunteers from the baseline examination was performed. The exact same conditions were used on baseline and evaluation recordings (Figure1).

### Data analysis

Despite the fact that six cineloops were recorded during data acquisition, only the four cineloops from the cardiac views were included in the analysis. Image analysis with regard to technical quality was performed by a blinded independent observer with a systematic approach based on methods described in previous studies [5,7]. Images were graded according to the following five aspects: 1) Anatomical presentation, 2) sector optimization (depth and angle), 3) gain adjustment, 4) image (resolution) sharpness, and 5) interpretational value. Items 1, 2, 4 and 5 received a numeral score on a 5-point scale and item number 3 received a numeral score on a 3 point scale. Details on each image grading aspect are shown in Table 1. A global image rating was calculated for all participants with a possible minimum score of 20 and a possible maximum score of 92. 

**Table 1:** Detailed description of aspects in the image analysis

**Anatomical presentation**				
Highest possible score was 5, one point was deducted for each of the errors in probe orientation mentioned below.				
Rotation	Angulation	Tilt	Thoracic position	
**Sector optimization (depth and angle width)**				
Highest possible score was 5, one or two points were deducted upon suboptimal sector as mentioned below.				
Small depth error	Large depth error	Small angle error	Large angle error	
(1 point)	(2 points)	(1 point)	(2 points)	
**Gain adjustment**				
Highest possible score was 3, points were deducted as mentioned below.				
Small undergain	Large undergain	Small overgain	Large overgain	
(1 point)	(2 points)	(1 point)	(2 points)	
**Image (resolution) sharpness**				
A score of 1 to 5 was given based on the scale below.				
1: No visible myocardium	2: Barely visible myocardium	3: Fairly visible myocardium	4: Visible Myocardium and endocardium	5: Perfect presentation of myo- and endocardium
**Interpretational value**				
A score of 1 to 5 was given based on the scale below. A score of 3 was perceived as minimum for images suitable for interpretation.				
1: No interpretation possible	2: Uncertain interpretation possible	3: Rough interpretation possbile	4: Interpretation possible	5: Interpretation and measurement possible

Results from the mental rotation test were graded as described in the original work [14] and expressed as a percentage of correct answers. Because of age homogeneity in the group no adjustment for age was made.

### Statistical analysis

Data was considered normally distributed after examination of the Q-Q plots. For comparison of baseline data and global image ratings a paired two-tailed t-test was used. Results from the metal rotation test and the data on correct probe orientation was compared with global image ratings using Pearson’s correlation. Level of significance was p = 0.05. Tests and calculations were carried out using Stata 11.0 software (StataCorp LP, Texas, USA). Data are presented as mean and confidence interval.

## Results

At baseline 86% of images obtained by novices were suitable for interpretation, on evaluation this was 93% (p = 0.005). 100% of images obtained by experts were suitable for interpretation. For novices, the mean global image rating on baseline examinations was 70.2 (68.0-72.4) and on evaluation 75.0 (72.9-77.0), p = 0.0002. In comparison, mean global image rating in the expert group was 89.8 (88.8-90.9), which was significantly higher than novices on evaluation (p < 0.0001). Detailed results from each category in the image analysis are shown in Table 2.

**Table 2:** Detailed results from the image analysis

	**Anatomical presentation**	**Sector optimization**	**Gain adjustment**	**Image sharpness**	**Interpretational value**
**Before intervention**					
**Mean**	3.40	4.01	2.69	3.72	3.70
**CI**	3.21-3.59	3.84-4.17	2.60-2.80	3.56-3.88	3.50-3.89
**After intervention**					
**Mean**	3.68	4.50	2.80	3.81	3.96
**CI**	3.50-3.86	4.38-4.62	2.70-2.89	3.68-3.94	3.78-4.14
**P-value**	0.002	<0.0001	0.086	0.25	0.008

**Figure 2:**
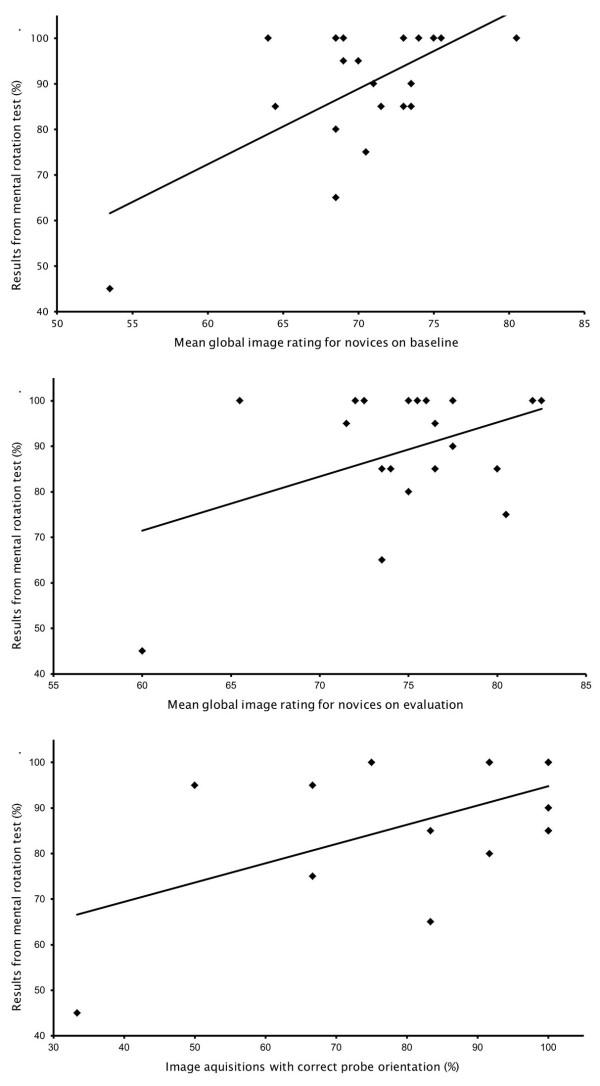
***Upper panel: *Scatter plot showing the relationship between visual spatial ability measured by a mental rotation test and mean global image rating for novices before intervention (Pearson 0.61, p = 0.0034).*** Middle panel:* Scatter plot showing the relationship between visual spatial ability measured by a mental rotation test and mean global image rating for novices after intervention (Pearson 0.44, p = 0.047). *Lower panel:* Scatter plot showing the relationship between visual spatial ability measured by a mental rotation test and the percentage of correct probe orientations on baseline (Pearson 0.56, p = 0.0084).

A correlation was found between results from the mental rotation test and the mean global image rating for novices on baseline (Figure2, *upper panel*) r = 0.61 (p = 0.0034) and evaluation (Figure2, *middle panel*) r = 0.44 (p = 0.047). Likewise, a moderate correlation was found between results from the mental rotation test and the ability to orientate the probe correctly (Figure2, *lower panel*) r = 0.56 (p = 0.0084). The mean global image rating at baseline correlated significantly with the ability to orientate the probe correctly r = 0.63 (p = 0.0020). Correct orientation of the probe improved from 87% before intervention to 99% after intervention. There was no significant difference between mean global image rating and previous or no previous experience in ultrasonography for novices on baseline (p = 0.39) or evaluation (p = 0.36). Likewise, no significant difference (p = 0.37) was found in proportion of examinations with correct probe orientation when comparing participants with previous and no previous experience in ultrasonography.

## Discussion

The data presented suggest that a very limited intervention is needed to improve the technical skills of inexperienced physicians to perform FATE. The 10 supervised examinations were performed as one-on-one training in approximately three hours and resulted in significant improvement of technical image quality. Adding the three hours of supervision to the two hours of introduction sums up to a total of 5 hours training after which the novices were able to produce images suitable for interpretation in 93% of all cases. In addition, all examinations by novices contained at least one image suitable for interpretation. Not surprisingly, experts generally received higher image scores. Higher image quality is especially relevant when performing diagnostic echocardiography including advanced measurements and Doppler methods whereas images with fewer details can be sufficient in more acute situations. With POC examinations in mind, the presented data suggest that physicians with a very limited amount of training can achieve ultrasound images with great importance for evaluation of immediate cardiopulmonary status.

One of the primary obstacles to overcome during training in FATE was correct orientation of the probe, a trait that could relate to the physicians inborn visual spatial ability. Physicians with high scores in the mental rotation test indeed had superior image ratings and better probe orientation on baseline examinations. However, the issue with correct probe orientation quickly resolves, as revealed by a very high degree of correct probe orientation on evaluation. There still remained a weak, but significant correlation between abilities in the mental rotation test and image rating after the intervention, although this was less distinct than during baseline examinations. One explanation for this finding could be that we found somewhat a ceiling effect of the mental rotation test which could influence the results and it is therefore difficult to draw any significant conclusions from the current data.

When comparing novices with previous experience in ultrasound examination and novices with no previous experience, no significant difference in image rating or correct probe orientation was found. A possible explanation could be that almost all previous ultrasound experience was from vascular access and ultrasound guided nerve blocks. These procedures are done with linear probes, and adjustments are primarily done in two dimensions. This contrasts the sector probes used for FATE examinations, where correct probe orientation requires adjustment in all the four dimensions mentioned in Table 2.

The aspect of time consumption was not addressed in this study. Participants were allowed a total of 9 minutes for acquisition of the six standard views in the FATE protocol. Previous studies have shown that with sole focus on image acquisition experienced examiners can perform the protocol in around 70 seconds [7].

Results from the current study indicate that physicians can acquire the ability to perform focused cardiac ultrasound following a very limited training. However, in order not to compromise examination quality we advocate that there be a system in place for continuing expert back-up and supervision, quality control, continuing education, and re-accreditation.

### Limitations

Baseline and post-intervention exanimations were performed on healthy volunteers with good image quality. This is often not the case in clinical situations, and the difference between novice and experts might be clearer in the clinical setting.

The image analysis is a subjective assessment and the scoring of subcategories has the potential for crossover when using a global score. The subjectivity was handled by using a blinded independent observer for the image analysis and possible issues concerning the global score was handled by showing results from all subcategories.

Disclosure of pathology and definition of cardiopulmonary status is the apparent objective when performing FATE. Previous studies have shown that inexperienced examiners can learn aspects of POC echocardiography with limited training [9,16,17]. However, this study did not focus on the ability to interpret the ultrasonic images. This issue requires further attention in future studies.

## Conclusions

Improvement of technical skills in FATE can be achieved with a limited intervention resulting in 93% of images achieved being suitable for clinical interpretation. Inborn visual spatial ability data did not seem to have significant impact on performance when acquiring technical skill in FATE, but further investigation is warranted. Previous experience with ultrasound in other fields did not influence the acquisition of technical skills in FATE.

## Abbreviations

POC: Point-of-care; FATE: Focus Assessed Transthoracic Echocardiography.

## Competing interests

The authors declare that they have no competing interests.

## Authors’ contribution

CF, PJ, DN, BE and ES participated in the design of the study. CF, PJ and DN carried out the data acquisition. CF and PJ performed the data analysis and CF performed the statistical analysis. CF and ES drafted the manuscript. All authors read, revised and approved the final manuscript.

## Pre-publication history

The pre-publication history for this paper can be accessed here:

http://www.biomedcentral.com/1472-6920/12/65/prepub

## References

[B1] Moore CL Copel JA Point-of-care ultrasonography N Engl J Med 2011 364 749 757 10.1056/NEJMra0909487 21345104

[B2] Sankoff J Keyes LE Emergency medicine resident education: making a case for training residents to perform and interpret bedside sonographic examinations Ann Emerg Med 1999 34 105 108 10.1016/S0196-0644(99)70281-7 10382004

[B3] Beagle GL Bedside diagnostic ultrasound and therapeutic ultrasound-guided procedures in the intensive care setting Crit Care Clin 2000 16 59 81 10.1016/S0749-0704(05)70097-X 10650500

[B4] Moore CL Gregg S Lambert M Performance, training, quality assurance, and reimbursement of emergency physician-performed ultrasonography at academic medical centers J Ultrasound Med 2004 23 459 466 15098862 10.7863/jum.2004.23.4.459

[B5] Jensen MB Sloth E Larsen KM Schmidt MB Transthoracic echocardiography for cardiopulmonary monitoring in intensive care Eur J Anaesthesiol 2004 21 700 707 15595582 10.1017/s0265021504009068

[B6] Jakobsen CJ Torp P Sloth E Perioperative feasibility of imaging the heart and pleura in patients with aortic stenosis undergoing aortic valve replacement Eur J Anaesthesiol 2007 24 589 595 10.1017/S0265021506002328 17462116

[B7] Frederiksen CA Juhl-Olsen P Larsen UT Nielsen DG Eika B Sloth E New pocket echocardiography device is interchangeable with high-end portable system when performed by experienced examiners Acta Anaesthesiol Scand 2010 54 1217 1223 10.1111/j.1399-6576.2010.02320.x 21039344

[B8] Nielsen DG Gotzsche O Sonne O Eika B The relationship between immediate relevant basic science knowledge and clinical knowledge: physiology knowledge and transthoracic echocardiography image interpretation Adv Health Sci Educ Theory Pract 2011 10.1007/s10459-011-9327-y 21952688

[B9] Martin LD Howell EE Ziegelstein RC Martire C Shapiro EP Hellmann DB Hospitalist performance of cardiac hand-carried ultrasound after focused training Am J Med 2007 120 1000 1004 10.1016/j.amjmed.2007.07.029 17976430

[B10] Martin LD Howell EE Ziegelstein RC Martire C Whiting-O’Keefe QE Shapiro EP Hellmann DB Hand-carried ultrasound performed by hospitalists: does it improve the cardiac physical examination? Am J Med 2009 122 35 41 10.1016/j.amjmed.2008.07.022 19114170

[B11] Hellmann DB Whiting-O’Keefe Q Shapiro EP Martin LD Martire C Ziegelstein RC The rate at which residents learn to use hand-held echocardiography at the bedside Am J Med 2005 118 1010 1018 10.1016/j.amjmed.2005.05.030 16164888

[B12] Bose R Matyal R Panzica P Karthik S Subramaniam B Pawlowski J Mitchell J Mahmood F Transesophageal echocardiography simulator: a new learning tool J Cardiothorac Vasc Anesth 2009 23 544 548 10.1053/j.jvca.2009.01.014 19303326

[B13] Bose RR Matyal R Warraich HJ Summers J Subramaniam B Mitchell J Panzica PJ Shahul S Mahmood F Utility of a transesophageal echocardiographic simulator as a teaching tool J Cardiothorac Vasc Anesth 2011 25 212 215 10.1053/j.jvca.2010.08.014 20974542

[B14] Vandenberg SG Kuse AR Mental rotations, a group test of three-dimensional spatial visualization Percept Mot Skills 1978 47 599 604 10.2466/pms.1978.47.2.599 724398

[B15] Peters M Laeng B Latham K Jackson M Zaiyouna R Richardson C A redrawn Vandenberg and Kuse mental rotations test: different versions and factors that affect performance Brain Cogn 1995 28 39 58 10.1006/brcg.1995.1032 7546667

[B16] Price S Via G Sloth E Guarracino F Breitkreutz R Catena E Talmor D Echocardiography practice, training and accreditation in the intensive care: document for the World Interactive Network Focused on Critical Ultrasound (WINFOCUS) Cardiovasc Ultrasound 2008 6 49 10.1186/1476-7120-6-49 18837986 PMC2586628

[B17] Vignon P Mucke F Bellec F Marin B Croce J Brouqui T Palobart C Senges P Truffy C Wachmann A Basic critical care echocardiography: validation of a curriculum dedicated to noncardiologist residents Crit Care Med 2011 39 636 642 10.1097/CCM.0b013e318206c1e4 21221001

